# Lethean Vapor

**Published:** 1847-03

**Authors:** 


### LETHEAN VAPOR.

We lately had an opportunity of witnessing the extraction of teeth from two gentlemen who had inhaled this vapor. They were patients of Mr. Goddard, and Mr. Somerby was present and assisted.

*Case* 1. A vigorous man, in or approaching middle life. A short time before the inhalation commenced, his pulse was 70 in a minute; full and strong. When about to be given, though he was not apparently alarmed, it rose to 112. After a short inhalation, less than a minute, the tube fell from his hand, and the operation was commenced. His pulse had then risen to 144. His pupils did not become dilated. From difficulty in the operation, it proved rather tedious; but he remained motionless, and made no manifestation of pain. In about two minutes from the time he passed into a state of insensibility, he suddenly emerged from it. His pulse was then 120, and in a quarter of an hour had fallen to 70. His intellectual faculties and feelings were quite natural immediately on awaking. He was unconscious of any pain, but said he had dreamed of some kind of crash.

*Case* 2. A young man had two fangs of different teeth extracted. Pulse before breathing the gas, 88, and increased but a few beats afterwards. No dilatation of the pupil. After the loss of the first fang, was aroused, and said he had felt the cutting of the gums, but not the extraction. Inhaled the gas a second time, and during the operation which followed, stretched himself out, and acted as he might have done if the vapor had not been breathed. On recovering, however, he declared that he had not felt any pain. The sensorial functions seemed to have been asleep, but the excito-motory awake.

